# Risk of Hypertension and Type 2 Diabetes in Relation to Changes in Alcohol Consumption: A Nationwide Cohort Study

**DOI:** 10.3390/ijerph19094941

**Published:** 2022-04-19

**Authors:** Jae Woo Choi, Euna Han, Tae Hyun Kim

**Affiliations:** 1 Community Care Research Center, Health Insurance Research Institute, National Health Insurance Service, Wonju 26464, Korea; jwchoi2695@hanmail.net; 2 College of Pharmacy, Yonsei Institute of Pharmaceutical Sciences, Yonsei University, Incheon 21983, Korea; eunahan@yonsei.ac.kr; 3 Department of Healthcare Management, Graduate School of Public Health, Yonsei University, Seoul 03722, Korea

**Keywords:** occasional moderate drinking, frequent binge drinking, hypertension, type 2 diabetes, Korean adults, longitudinal cohort study

## Abstract

Little is known about the risk of hypertension and type 2 diabetes in relation to changes in frequency and amount of alcohol consumption. This study investigated associations between changes in alcohol consumption and the risk of both conditions. This study included 96,129 individuals without hypertension and/or diabetes mellitus aged ≥ 20 years between 2006 and 2008, with follow-up until 31 December 2015. This study identified 29,043 and 18,784 incident cases of hypertension and type 2 diabetes, respectively, during an average follow-up period of 6.2 ± 2.6 and 6.9 ± 1.9 years. This study measured changes in frequency and amount of alcohol consumption using standardized self-administered questionnaires over approximately 2 years. Hazard ratio (HR) and 95% confidence interval (CI) were calculated for the respective risks of the two conditions. Repeated occasional or frequent binge drinking was associated with an increased risk of hypertension (HR: 1.16 or 1.32; 95% CI: 1.11, 1.21 or 1.16, 1.51) and type 2 diabetes (HR: 1.14 or 1.36; 95% CI: 1.09, 1.20 or 1.17, 1.58) compared with continuous nondrinking. Reductions as well as increases in frequency of alcohol consumption among binge drinkers were associated with higher hypertension (HR: 1.29 or 1.30; 95% CI: 1.11, 1.49 or 1.13, 1.49) and type 2 diabetes (HR: 1.26 or 1.56; 95% CI: 1.06, 1.49 or 1.34, 1.81) risk. This study demonstrated that repeated binge drinking, even with a reduction of weekly alcohol consumption frequency, was associated with a higher risk of hypertension and type 2 diabetes.

## 1. Introduction

Cardiometabolic diseases are common, presenting a considerable challenge to many countries [1]. The global age-standardized prevalence of hypertension is increasing (25.9% in 2000 to 30.8% in 2010) [2], and the worldwide prevalence of diabetes mellitus in 2019 was 9.3%; this prevalence is expected to rise to 10.9% (700 million) by 2045 [3]. Given that hypertension and diabetes mellitus are leading causes of premature death and are significant risk factors for kidney and cardiovascular diseases [4,5,6], prevention of these conditions is an important worldwide public health priority. Furthermore, globally, the number of confirmed cases of COVID-19 is 476.4 million, including 6.1 million deaths according to the World Health Organization (WHO), the spread of COVID-19 is predicted to increase worldwide [7], and diabetes and hypertension seem to negatively affect the clinical course and disease outcome in patients with COVID-19 [8].

Alcohol consumption is one of the largest public health challenges facing modern society and is ranked as the world’s third largest risk factor for disease burden [9]. An increasing trend has been observed worldwide for alcohol consumption per capita over time from 5.5 L in 2005 to 6.4 L in 2016. In Korea, people consumed a remarkable amount of alcohol, with an average of 10.2 L per year in 2016, the highest level among Asian countries after Laos with 10.4 L [10]. A recent study had demonstrated that excessive alcohol use increases all-cause and cause-specific mortality risks in both sexes. The all-cause mortality risk for men (hazard ratio (HR) 95% confidence interval (CI): 1.55 (1.23, 1.96)) with alcohol intakes above the recommended maximum intake was similar to that for women (HR [95% CI]: 1.38 (1.05, 1.83)) [11]. Furthermore, binge drinking has been associated with an increased risk of hypertension [12,13] and diabetes mellitus [14,15], although the protective health effects of moderate alcohol consumption remain controversial [16,17].

These studies had used measurements of alcohol consumption at only one time point (baseline), with no repeated measurements during follow-up. The previous studies have established pooled nondrinkers as the reference group. However, nondrinkers, who include both never and former drinkers, are far from homogeneous. In particular, former drinkers are noteworthy, with poorer health and higher mortality rates than moderate drinkers and never drinkers [18]. Therefore, many existing findings may have overestimated the degree of risk reduction by contrasting drinkers to less healthy nondrinkers including former drinkers [19]. In a meta-analysis examining the association between alcohol drinking and all-cause mortality, the risk reductions were attenuated when data were limited to studies that eliminated former drinkers from the reference group [20]. Furthermore, many people change their levels of alcohol consumption throughout life and these changes might affect the risk of hypertension and type 2 diabetes. Although several studies explored the association of repeatedly measured alcohol consumption with risk of hypertension [21,22] and type 2 diabetes [23,24,25], none of these studies had considered changes in the frequency and amount of alcohol consumption at the same time due to their relatively small samples; thus, little is known about the effect of the changes in alcohol consumption on these conditions, particularly in adults without hypertension and/or diabetes mellitus.

This study investigated the association between alcohol consumption at baseline as well as changes in alcohol consumption with subsequent risk of hypertension and type 2 diabetes among adults using large, nationwide cohort data.

## 2. Methods

### 2.1. Data and Study Sample

We employed a concrete cohort profile from the National Health Insurance Service–National Sample Cohort (NHIS–NSC) [26]. In brief, a nationwide representative sample of one million individuals (500,268 men; 499,732 women) had been established in 2006 from the overall Korean population. The NHIS–NSC contains information on the sample’s sociodemographic characteristics, records from all medical visits, results of national health screening, and death dates from 2002 to 2015.

**Figure 1:**
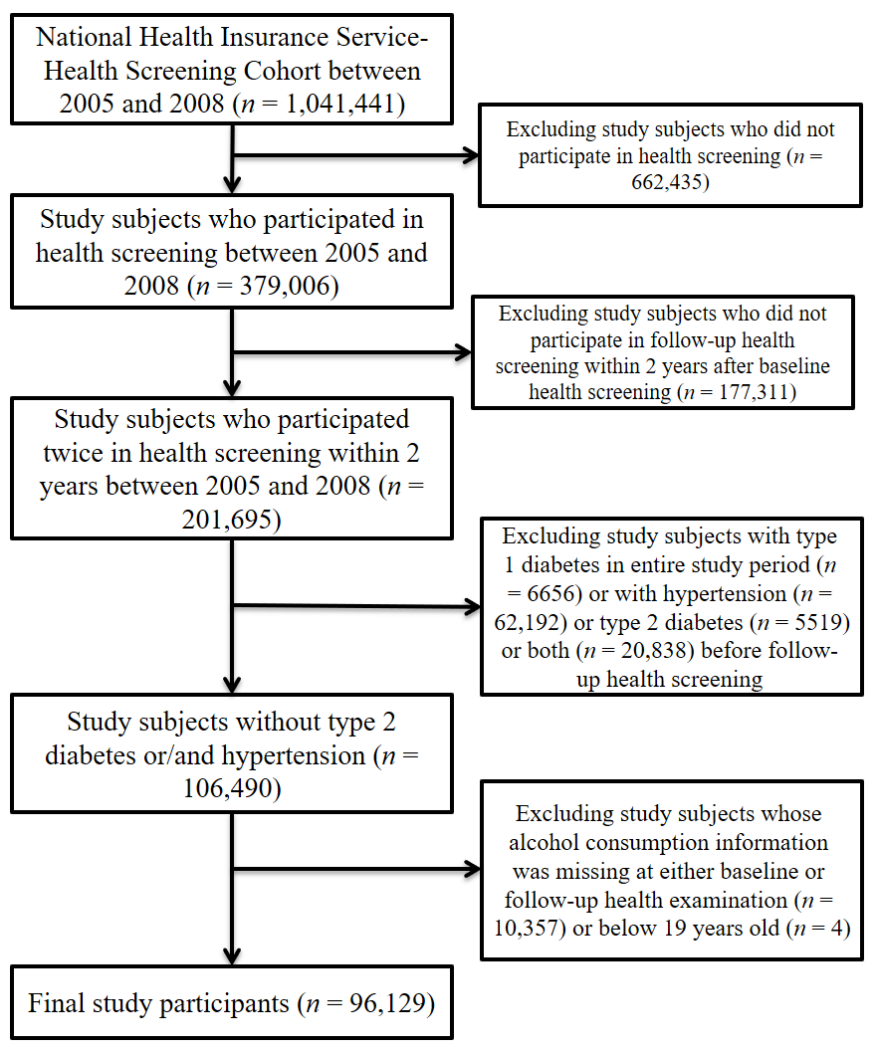
Flow chart of the study participants.

Among the 1,041,441 study participants included in the NHIS–NSC between 2005 and 2008, we excluded the following individuals: those who did not have information on national health screening (*n* = 662,435), as given alcohol consumption was surveyed in this screening; those who did not undergo a follow-up national health screening (2006–2008) within 2 years after a baseline national health examination (2005–2007) (*n* = 177,311) to measure changes in alcohol consumption. This study also excluded study participants who, during the entire study period, had type 1 diabetes (*n* = 6656), hypertension (*n* = 62,192), type 2 diabetes (*n* = 5519), or both hypertension and type 2 diabetes (*n* = 20,838) prior to the follow-up health screening. Finally, we excluded study participants whose alcohol consumption information was missing either at baseline or during the follow-up health screening (*n* = 10,357) or who were younger than 19 years of age (*n* = 4). Ultimately, 96,129 individuals were selected as the final participants in this study (Figure 1).

### 2.2. Measurements for Hypertension and Type 2 Diabetes

The study’s dependent variables were the incidence of hypertension and type 2 diabetes. Hypertension was defined as high blood pressure (BP) (systolic BP ≥ 140 mm Hg and/or diastolic BP ≥ 90 mm Hg) or a history of prescriptions for antihypertensive medication. Type 2 diabetes was defined by any of the following conditions: type 2 diabetes (*The International Classification of Disease 10th Revision* (ICD-10) code: E11–E14) diagnosed by a physician at least twice within 6 months; fasting blood glucose level of ≥7 mmol/L (126 mg/dL); or a history of prescriptions for oral hyperglycemic medication or insulin. Blood pressure was measured once on the right arms of seated participants who had rested for >5 min using automatic blood pressure monitor, and if the blood pressure was elevated and classified as hypertensive, a second blood pressure was measured [27]. Blood samples were collected from each participant after overnight fasting. The laboratory examinations included assessments of serum total cholesterol, low-density lipoprotein cholesterol, high-density lipoprotein cholesterol, triglycerides, and fasting glucose. All the biochemical analyses were carried out using standardized laboratory methods [28].

### 2.3. Measurements for Alcohol Consumption

Alcohol consumption was evaluated based on information from individuals’ responses to standardized self-administered questionnaires during the national health screening [29]. Participants were asked the frequency of alcohol consumption per week and the average amount of alcohol consumption per occasion in relation to Korea’s most popular alcoholic beverage, “*soju*.” Based on the data collected, standard drinks were calculated as following: number of standard drinks = 0.785 (the specific gravity of ethyl alcohol) × 360 mL (volume of container in liters) × 20% (percentage of alcohol volume). For example, a bottle of *soju* is equivalent to 4 standard drinks. Binge drinking was defined as the consumption of ≥4 standard drinks per occasion, approximately consistent with the amount proposed by the National Institute on Alcohol Abuse and Alcoholism Council [30]. According to the frequency of alcohol consumption, the study participants were classified into nondrinking, occasional drinking (≤5 times/week), and frequent drinking (>5 times/week) groups [31]. The participants were further categorized according to the alcohol consumption patterns combining frequency and amount of alcohol consumption as follows: “nondrinking,” “occasional moderate drinking (≤5 times/week and <4 standard drinks/occasion),” “frequent moderate drinking (>5 times/week and <4 standard drinks/occasion),” “occasional binge drinking (≤5 times/week and ≥4 standard drinks/occasion),” and “frequent binge drinking (>5 times/week and ≥4 standard drinks/occasion)” groups.

### 2.4. Measurement for Potential Confounding Factors

Potential covariates in this study were sex, age, BMI, systolic BP, diastolic BP, fasting glucose, total cholesterol, smoking status, exercise habits, nutrition intake behaviors, household income, location of residence, family history of hypertension, family history of diabetes, and comorbidities. BMI was estimated as the weight in kilograms divided by the squared height in meters and was classified into 5 groups as follows: ≤18.4 kg/m^2^ (underweight); 18.5–22.9 kg/m^2^ (normal weight); 23.0–24.9 kg/m^2^ (overweight); 25.0–29.9 kg/m^2^ (class I obese); and ≥30.0 kg/m^2^ (class II obese), based on World Health Organization recommendations for Asian populations [32]. Fasting glucose and total cholesterol were estimated by blood sampling following overnight fasting. Smoking status was categorized as nonsmoking, ex-smoking, or current smoking. Exercise was defined as exercising at least once a week. Nutrition intake behaviors were classified into meat as a staple food (those who primarily eat meat), vegetables as a staple food (those who primarily eat vegetables), and mixed intake (those who eat meat and vegetables equally). Household income was classified as follows: high (81st–100th percentile); middle (41st–80th percentile); or low (0–40th percentile). Location of residence was defined as metropolitan (capital), urban (local government with ≥1 million people), or rural (other). All covariates excluding comorbidities were estimated at the date of the follow-up health examination. The comorbidities were dyslipidemia (ICD-10 code: E78), stroke (ICD-10 codes: I60–I63), ischemic heart disease (ICD-10 codes: I20–I25), and cancer (ICD-10 codes: C00–C99), and they were estimated using information on records from all medical visits before the follow-up health screening.

### 2.5. Statistical Analyses

We estimated the general characteristics of the study participants at baseline and follow-up health examination using mean (standard deviation) for continuous variables and *n* (%) for categorical variables. For each individual, the length of follow-up was estimated in days, and all study participants were followed from the date of the follow-up health examination until the onset of hypertension or type 2 diabetes, death by any cause, or until the end of 2015, whichever occurred first.

We used Cox proportional hazards regression models to assess the association of alcohol consumption at baseline and changes in alcohol consumption with risk of hypertension and type 2 diabetes. The adjusted hazard ratio (HR) and 95% CI for both conditions in the Cox regression models were measured separately. In analysis for association between alcohol consumption and risk of hypertension, we adjusted sex, age, BMI, systolic blood pressure, diastolic blood pressure, total cholesterol, family history of hypertension, smoking status, exercise, nutrition intake behaviors, household income, residential area, and comorbidities as potential covariates. In analysis for association between alcohol consumption and risk of type 2 diabetes, we adjusted sex, age, BMI, fasting glucose, total cholesterol, family history of diabetes, smoking status, exercise, nutrition intake behaviors, household income, residential area, and comorbidities as potential covariates. First, we assessed the association between alcohol consumption at the baseline health examination and the risk of hypertension and type 2 diabetes and included the covariates estimated at the baseline health screening in the Cox regression models. Second, we analyzed the association between changes in alcohol consumption and the risk of both conditions and used covariates measured at the follow-up health examination in the Cox regression models.

All data extractions and statistical analyses were performed using SAS v9.4 (SAS Institute Inc., Cary, NC, USA). Proportional hazards assumptions were assessed statistically and satisfied for all models. The Yonsei University Institutional Review Board approved this study, and the requirements for informed consent were waived because the NHIS–NSC data were established following anonymization by guidelines for rigorous confidentiality.

## 3. Results

We identified 29,043 and 18,784 incident cases of hypertension and type 2 diabetes during an average follow-up period of 7.7 ± 2.6 and 8.4 ± 1.9 years from baseline and 6.2 ± 2.6 and 6.9 ± 1.9 years from the follow-up health examination, respectively. The average interval between baseline and follow-up health screening was 1.5 ± 0.6 years. Table 1 shows the general characteristics of the study participants who received two consecutive biennial national health examinations in 2005–2008. At the follow-up health screening, the mean age (standard deviation) was 42.8 (13.0) years; 50,298 (52.3%) of the participants were men. Approximately half of the participants at the baseline and follow-up health examination periods responded that they were nondrinkers (50.5% at baseline and 49.7% at follow-up). The proportion of occasional moderate drinking, frequent moderate drinking, occasional binge drinking, and frequent binge drinking at the follow-up health screening was 16.1%, 0.5%, 32.5%, and 1.3%, respectively. The other characteristics of the study participants who underwent the two consecutive biennial health screenings from 2005 to 2008 are presented in Table 1.

Table 2 provides the HR and 95% CI for the risk of developing hypertension or type 2 diabetes by alcohol consumption status at baseline. After adjusting for sex, age, BMI, systolic BP (for hypertension only), diastolic BP (for hypertension only), fasting glucose (for type 2 diabetes mellitus only), total cholesterol, family history of hypertension (for hypertension only), family history of diabetes mellitus (for type 2 diabetes only), smoking status, exercise, nutrition intake behaviors, household income, residential area, and comorbidities, an increased risk of hypertension and type 2 diabetes was observed in occasional binge drinking (HR, 1.11 and 1.10; 95% CI, 1.07–1.15 and 1.06–1.15, respectively) and frequent binge drinking (HR, 1.24 and 1.21; 95% CI, 1.13–1.36 and 1.09–1.35, respectively) compared with the nondrinking, whereas there was no significant association with occasional moderate drinking (HR, 0.99 and 0.98; 95% CI, 0.96–1.03 and 0.93–1.02, respectively) or frequent moderate drinking (HR, 1.01 and 0.98; 95% CI, 0.88–1.16 and 0.83–1.15, respectively) with the risk of hypertension and type 2 diabetes compared with nondrinking.

**Figure 2:**
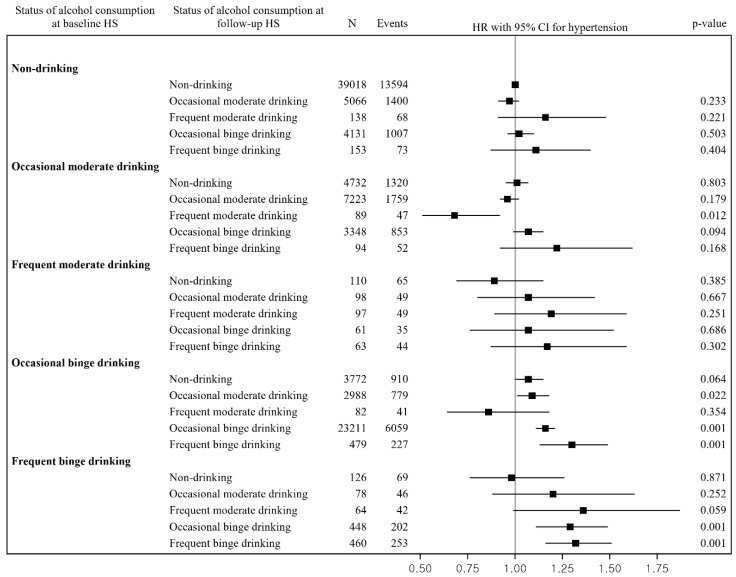
Relationship between changes in alcohol consumption and risk of hypertension. Abbreviations: CI = confidence interval; HR = hazard ratio; HS = health screening. The HR and 95% CI were estimated after adjusting for sex, age, BMI, systolic blood pressure, diastolic blood pressure, total cholesterol, family history of hypertension, smoking status, exercise, nutrition intake behaviors, household income, residential area, and comorbidities.

Figure 2 indicates the adjusted HR (95% CI) for risk of hypertension in relation to changes in alcohol consumption compared with continuous nondrinking, which was 0.68 (0.51–0.92) for changes from occasional moderate drinking at baseline to frequent moderate drinking at the follow-up; 1.09 (1.01–1.18) for changes from occasional binge drinking at baseline to occasional moderate drinking at the follow-up; 1.16 (1.11–1.21) for repeated occasional binge drinking; 1.30 (1.13–1.49) for changes from occasional binge drinking at baseline to frequent binge drinking at the follow-up; 1.29 (1.11–1.49) for changes from frequent binge drinking at baseline to occasional binge drinking at the follow-up; and 1.32 (1.16–1.51) for repeated frequent binge drinking.

**Figure 3:**
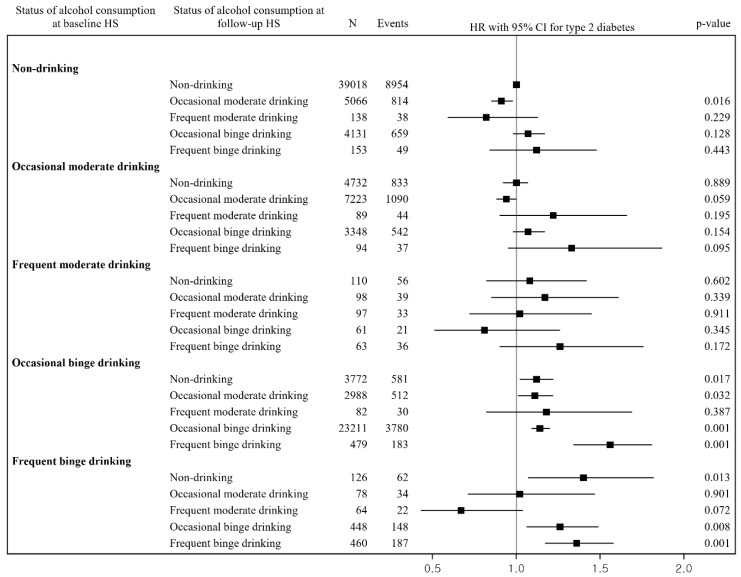
Relationship between changes in alcohol consumption and risk of type 2 diabetes. Abbreviations: CI = confidence interval; HR = hazard ratio; HS = health screening. The HR and 95% CI were estimated after adjusting for sex, age, BMI, fasting glucose, total cholesterol, family history of diabetes, smoking status, exercise, nutrition intake behaviors, household income, residential area, and comorbidities.

Figure 3 shows the adjusted HR (95% CI) for the risk of type 2 diabetes in relation to changes in alcohol consumption compared with continuous nondrinking, which was 0.91 (0.85–0.98) for changes from nondrinking at baseline to occasional moderate drinking at the follow-up; 1.12 (1.02–1.22) for changes from occasional binge drinking at baseline to nondrinking at the follow-up; 1.11 (1.01–1.22) for changes from occasional binge drinking at baseline to occasional moderate drinking at the follow-up; 1.14 (1.09–1.20) for repeated occasional binge drinking; 1.56 (1.34–1.81) for changes from occasional binge drinking at baseline to frequent binge drinking at the follow-up; 1.40 (1.07–1.82) for changes from frequent binge drinking at baseline to nondrinking at the follow-up; 1.26 (1.06–1.49) for changes from frequent binge drinking at baseline to occasional binge drinking at the follow-up; and 1.36 (1.17–1.58) for repeated frequent binge drinking.

## 4. Discussion

This nationwide cohort study verified that occasional or frequent binge drinking was associated with an increased risk of hypertension and type 2 diabetes compared with nondrinking. This study also demonstrated that repeated occasional or frequent binge drinking was associated with an increased risk of hypertension and type 2 diabetes compared with continuous nondrinking. A reduction (frequent to occasional) as well as an increase (occasional to frequent) in weekly frequency in binge drinkers was associated with a higher risk of both conditions.

Several biological mechanisms have been proposed to explain the association between alcohol consumption and hypertension and type 2 diabetes. Alcohol is likely to increase BP by opposing relaxation of the endothelium, thus inhibiting the production of nitric oxide, which has anti-oxidative effects [33]. Alcohol consumption is also correlated with type 2 diabetes through its effects on insulin resistance, changes in alcohol metabolite levels, and anti-inflammatory effects [34,35].

Our findings indicating an increased risk of hypertension and type 2 diabetes in occasional or frequent binge drinking at baseline are consistent with previous results. Yoo et al. (2019) had found that high alcohol consumption (≥30 g/day for men, ≥15 g/day for women) was associated with increased risk of incident hypertension (HR: 1.95, 95% CI: 1.29, 2.94 for men; HR: 1.95, 95% CI: 1.10, 3.46 for women) [13]. Okubo et al. (2013) had shown a positive association between alcohol consumption and the risk of hypertension by both sex and age group [12]. These studies had calculated the daily amount of alcohol consumption by combining information on weekly frequency and amount per occasion of alcohol consumption; however, there is no evidence considering weekly frequency and amount per occasion of alcohol consumption separately. Our results suggest that the amount per occasion rather than weekly frequency of alcohol consumption is an important factor for the development of both conditions.

When assessing changes in alcohol consumption, Jung et al. (2020) had used three different measures of alcohol consumption (baseline, most recent, and cumulative average), showing that consuming ≥30 mL/d of alcohol was associated with a risk of hypertension among men (incidence rate ratio (IRR): 1.72, 95% CI: 1.20, 2.48 for baseline; IRR: 1.69, 95% CI: 1.23, 2.33 for the most recent; IRR: 1.49, 95% CI: 1.09, 2.03 for cumulative average) [21]. Lui et al. (2020) had used a time-varying variable of alcohol consumption and revealed that elevated risks for hypertension were found for women drinking >14 drinks/week (adjusted odds ratio (AOR): 1.57, *p* = 0.023) and for men engaged in risky drinking (15−28 drinks/week) (AOR: 1.64, *p* = 0.016) [22]. The present study, along with previous findings, suggests that repeated occasional or frequent binge drinking is associated with an increased risk of hypertension.

For the effects changes in alcohol consumption have on the risk of type 2 diabetes, Joosten et al. (2011) had measured alcohol consumption between two time points over 4 years, showing that light drinkers who increased their intake to moderate levels (5.0–29.9 g/day) had a significantly lower risk of type 2 diabetes mellitus (HR: 0.75, 95% CI: 0.62, 0.90) compared with stable light drinkers (0–4.9 g/day) [23]. In addition to an increased risk of type 2 diabetes among binge drinkers, our findings showed that changes from occasional or frequent binge drinking to alcohol abstinence was associated with a higher risk of type 2 diabetes, which was consistent with previous studies. He et al. (2019) had examined the increase or decrease in alcohol consumption over 9 years and found that, for men with alcohol intake ≥ 7 drinks/week at baseline, reducing alcohol intake was associated with higher risk of diabetes mellitus (HR: 1.12, 95% CI: 1.02, 1.23) [24]. Meanwhile, Han et al. (2019) had created trajectories of alcohol consumption and indicated that two trajectories in men showing a reduction to moderate or light levels after heavy alcohol consumption during early adulthood were significantly associated with increased risk of type 2 diabetes mellitus (HR: 1.66, 95% CI: 1.18, 2.33; HR: 1.93, 95% CI: 1.01, 3.70) [25]. These results were partially supported by previous literature, which had found that life course drinking intensity independent of current alcohol consumption is significantly related to a higher prevalence of metabolic syndrome [36]. However, our understanding of why a significant association between alcohol abstinence and hypertension incidence was not found is limited. Further investigations to understand how and why adults make changes in lifestyle would help explain these findings.

Several limitations warrant consideration in this study. First, the information on alcohol consumption was collected from a self-reported questionnaire, which is subject to recall and reporting bias. Second, this study measured changes in alcohol consumption among individuals who underwent a follow-up national health screening within 2 years after a baseline national health examination, because Korean adults are requested to participate the national health screening annually (only manual workers) or biannually. Thus, the time interval for changes in alcohol consumption may be relatively short, indicating that approximately 28% of the total sample has changed drinking behaviors within 2 years. Further research is required for risk of hypertension and type 2 diabetes in relation to changes in alcohol consumption over longer periods. Third, since the analytic sample is about 10% of the original sample from NHIS, an issue may be raised as to whether the final sample can be representative of the overall Korean population. We confirmed that the prevalence of obesity, current smoking, and alcohol consumption was comparable with that reported in the Korea National Health and Nutrition Examination Survey in Korea [37]. Fourth, some factors (e.g., hereditary, sedentarism) that could influence the appearance of these two diseases were not adjusted in the statistical models due to the limitation of our data. Finally, this study included only Korean adults; thus, further research is needed to assess changes in alcohol consumption in relation to the development of both conditions in other ethnicities and countries.

## 5. Conclusions

In conclusion, this study demonstrated that repeated occasional or frequent binge drinking was associated with an increased risk of hypertension and type 2 diabetes compared with continuous nondrinking. Furthermore, a reduction as well as an increase in weekly frequency among binge drinkers was associated with a higher risk of both conditions. This study suggests that the amount per occasion rather than the weekly frequency of alcohol consumption is an important factor for the development of hypertension and type 2 diabetes. Future research is needed on the effect of repeated binge drinking on severe diseases (cancer, cardiovascular diseases) and mortality.

## Figures and Tables

**Table 1:** General characteristics of study participants who received 2 consecutive biennial national health examinations from 2005–2008.

Variables		Baseline Health Screening (2005–2007)		Follow-Up Health Screening (2006–2008)	
		*n*	%	*n*	%
Total		96,129	100.0	96,129	100.0
Status of alcohol consumption					
	Nondrinking	48,506	50.5	47,758	49.7
	Occasional moderate drinking	15,486	16.1	15,453	16.1
	Frequent moderate drinking	429	0.4	470	0.5
	Occasional binge drinking	30,532	31.8	31,199	32.5
	Frequent binge drinking	1176	1.2	1249	1.3
Men		50,298	52.3	50,298	52.3
Age (years), mean ± SD		41.3	12.8	42.8	13.0
BMI (kg/m^2^)					
	≤18.5	5162	5.4	4917	5.1
	18.5–23	44,824	46.6	43,841	45.6
	23–25	22,741	23.7	22,860	23.8
	25–30	21,592	22.5	22,454	23.4
	≥30	1796	1.9	2046	2.1
SBP (mm Hg), mean ± SD		117.1	12.9	117.4	12.8
DBP (mm Hg), mean ± SD		73.3	8.8	73.3	8.7
Fasting glucose (mg/dL), mean ± SD		89.9	13.4	91.0	13.7
Total cholesterol (mg/dL), mean ± SD		188.7	35.2	190.1	36.4
Smoking status					
	Nonsmoking	63,408	66.0	63,750	66.3
	Ex-smoking	7925	8.2	8038	8.4
	Current smoking	23,904	24.9	23,681	24.6
Exercise		44,262	46.0	46,154	48.0
Nutrition intake behaviors					
	Meat as staple food	6337	6.6	6269	6.5
	Vegetables as staple food	16,584	17.3	16,124	16.8
	Mixed intake	72,577	75.5	73,380	76.3
Household income					
	Low	32,951	34.3	28,726	29.9
	Middle	41,655	43.3	43,826	45.6
	High	21,523	22.4	23,577	24.5
Residential area					
	Metropolitan	18,153	18.9	18,057	18.8
	Urban	26,177	27.2	25,887	26.9
	Rural	51,799	53.9	52,185	54.3
Family history of diabetes		6732	7.0	7434	7.7
Family history of hypertension		7063	7.3	8148	8.5
Comorbidities					
	Dyslipidemia	10,979	11.4	15,305	15.9
	Stroke	1060	1.1	1544	1.6
	Ischemic heart disease	4133	4.3	5575	5.8
	Cancer	3086	3.2	4122	4.3

Abbreviations: SD = standard deviation; BMI = body mass index; SBP = systolic blood pressure; DBP = diastolic blood pressure.

**Table 2:** The risk of developing hypertension and type 2 diabetes in relation to alcohol consumption.

Variable		No. of Study Participants	Person-Years	Cases	HR	95% CI		*p*-Value
**Hypertension ^†^**								
	Nondrinking	48,506	367,181	16,142	1.00			
	Occasional moderate drinking	15,486	122,348	4031	0.99	0.96	1.03	0.677
	Frequent moderate drinking	429	2695	242	1.01	0.88	1.16	0.917
	Occasional binge drinking	30,532	238,312	8016	1.11	1.07	1.15	<0.001
	Frequent binge drinking	1176	7682	612	1.24	1.13	1.36	<0.001
**Type 2 diabetes ^‡^**								
	Nondrinking	48,506	406,556	10,514	1.00			
	Occasional moderate drinking	15,486	131,642	2546	0.98	0.93	1.02	0.290
	Frequent moderate drinking	429	3236	185	0.98	0.83	1.15	0.798
	Occasional binge drinking	30,532	254,385	5086	1.10	1.06	1.15	<0.001
	Frequent binge drinking	1176	8966	453	1.21	1.09	1.35	<0.001

Abbreviations: HR = hazard ratio; CI = confidence interval. ^†^ HR and 95% CI were estimated after adjusting for age, BMI, systolic blood pressure, diastolic blood pressure, total cholesterol, family history of hypertension, smoking status, exercise, nutrition intake behaviors, household income, residential area, and comorbidities. ^‡^ HR and 95% CI were estimated after adjusting for age, BMI, fasting glucose, total cholesterol, family history of diabetes, smoking status, exercise, nutrition intake behaviors, household income, residential area, and comorbidities.

## Data Availability

Data was obtained from a third party and are not publicly available.
